# *Panax ginseng* Polysaccharide Protected H9c2 Cardiomyocyte From Hypoxia/Reoxygenation Injury Through Regulating Mitochondrial Metabolism and RISK Pathway

**DOI:** 10.3389/fphys.2018.00699

**Published:** 2018-06-15

**Authors:** Yi-Han Zuo, Quan-Bin Han, Geng-Ting Dong, Rui-Qi Yue, Xue-Cong Ren, Jian-Xin Liu, Liang Liu, Pei Luo, Hua Zhou

**Affiliations:** ^1^State Key Laboratory of Quality Research in Chinese Medicine, Faculty of Chinese Medicine, Macau University of Science and Technology, Macau, China; ^2^International Institute of Translational Chinese Medicine, Guangzhou University of Chinese Medicine, Guangzhou, China; ^3^School of Chinese Medicine, Hong Kong Baptist University, Hong Kong, China; ^4^College of Pharmacy, Hunan University of Medicine, Huaihua, China

**Keywords:** *Panax ginseng*, acidic polysaccharides, cardioprotection, mitochondrial metabolism, RISK pathway

## Abstract

**Background and Objective:** Ischemic heart disease (IHD) has been the major issue of public health. *Panax ginseng* (ginseng) has been verified as an effective traditional Chinese medicines and exerted cardioprotective effect. This study aimed to investigate the polysaccharide fraction of ginseng on hypoxia/reoxygenation (H/R) injury in cardiomyocytes and the underlying mechanisms.

**Methods:** Ginseng was extracted by ethanol and fractionated by high-speed counter current chromatography (HSCCC) and column separation. The cardioprotective effect was evaluated in H9c2 cardiomyocytes underwent H/R treatment. The cell viability, apoptosis and mitochondrial respiration were examined.

**Results:** An acid polysaccharides fraction of ginseng (AP1) was identified the most effective fraction in protecting cardiomyocytes from H/R injury. AP1 restored the mitochondrial function by maintaining mitochondrial membrane potential (MMP), blocking the release of cytochrome C, and increasing the ATP generation and oxygen consumption rate (OCR) of cardiomyocytes. Meanwhile, AP1 induced the expression of glucocorticoid receptor (GR) and estrogen receptor (ER) which further activated reperfusion injury salvage kinase (RISK) pathway. Finally, AP1 increased nitric oxide (NO) production and regulated endothelial function by increasing endothelial NO synthase (eNOS) expression and decreasing inducible NOS (iNOS) expression in H/R injury.

**Conclusion:** The results suggested that AP1 exerted a protective effect in myocardial H/R injury mainly through maintaining myocardial mitochondrial function, thereby inhibiting myocardial H/R caused apoptosis and increasing the expressions of GR and ER, which in turn mediated the activation of RISK pathway and eNOS-dependent mechanism to resist the reperfusion injury.

## Introduction

According to the reports of World Health Organization [WHO] in 2014, about 7.4 million people died of IHD, making IHD the No. 1 human killer (World Health Organization, 2014). The main factor leading to damage of myocardium in IHD is the deprivation of nutrients and oxygen caused by coronary occlusion, and the degree and duration of arterial obstruction decide the damage severity. Timely reperfusion can be used to reduce the mortality in the short term effectively, but in the long term, it can trigger cardiac remodeling as the reperfusion process is accompanied with additional oxidative stress and cardiomyocyte inflammation, etc. (Moens et al., 2005; Yellon and Hausenloy, 2007; Hausenloy and Yellon, 2013). Thus, to improve patients’ prognosis, it is important to prevent or limit the damage to the myocardium in the early phase of reperfusion.

For the prevention and control of reperfusion injury, functional foods would be a suitable choice because of less adverse effects when they are taken regularly (Shahidi, 2004; Castro et al., 2005). In TCM system, it is believed that food and medicine originate from the similar source but with different applications and purposes. Ginseng, an herbal root of *Panax* ginseng C.A. Meyer, has over 2,000 years history of extensive uses as TCM and functional supplements (Liu and Xiao, 1992; Attele et al., 1999; Lu et al., 2009). Numerous experimental evidences have suggested that ginseng extract exerts therapeutic effects with multiple cardiovascular activities, such as reducing myocardial infract size, regulating blood circulation and improving lipid profiles (Vogler et al., 1999; Kim and Park, 2003; Ahn et al., 2011; Zhou et al., 2011). Many of the pharmacological actions of ginseng extract are produced by ginsenosides which belong to a common type of glycosides, and have been demonstrated by intensively studied to possess a pivotal role in the pharmacological activities of ginseng (Chen, 1996; Lu et al., 2009; Jiang et al., 2014; Lee and Kim, 2014). When compared to the effect of ginsenosides, ginseng polysaccharides are largely underappreciated or even discarded. For instance, in modern industrialized TCM preparation, polysaccharides are commonly removed as impurities to reduce the dosage amounts of final products. Nevertheless, polysaccharides, obtained from the water-soluble fraction of ginseng, have been demonstrated with immuneregulatory, anti-oxidative, and anti-cancer effects, etc (Lim et al., 2004; Sun et al., 2014; He et al., 2015). Several reports have showed that polysaccharides isolated from *Aralia elata* (Zhang et al., 2013a,b), *Ophiopogon japonicas* (Shi et al., 2015; Wang et al., 2015), and *Salvia miltiorrhiza* had significant cardioprotective effects (Song et al., 2013; Chang et al., 2016). However, the effects of ginseng polysaccharides on cardiac injury have not been reported yet. Therefore, this study aimed to isolate the polysaccharides from ginseng by bioassay guided separation and to investigate whether it has cardioprotective effect and then explore the underlying mechanisms of cardioprotective effect of this substances.

## Materials and Methods

### Raw Materials and Extraction of Ginseng

The root of *P. ginseng* was purchased from the wholesale market in Jinlin Province, China. The quality of *P. ginseng* has been certified to meet the requirements of Hong Kong Standard of Chinese Materia Medica and Chinese Pharmacopeia. Samples were stored in the laboratory in the dry environment until use, and the voucher specimens of the whole roots were stored in the School of Chinese Medicine of Hong Kong Baptist University. The ginseng extract (RSE) was prepared by ethanol extraction, a well-developed and widely accepted approach for preparation of the ginseng extract, according to our previous report (Zhou et al., 2011).

### Chemicals, Reagents, and Cell

The Millipore MILLI Q-Plus system (Bedford, MA, United States) was used to prepare deionized water. ACS grade methanol, chloroform, ethyl acetate and butanol were purchased from ACS Chemical, Inc. (Point Pleasant, NJ, United States). ACS grade ethanol, phenol, concentrated sulfuric acid, ammonia water, and sodium hydroxides were purchased from Merck (Darmstadt, Germany). TFA and ammonium acetate were bought from Riedel-de Haen (Morristown, NJ, United States). HPLC grade acetonitrile was purchased from RCL Labscan Limited (Bangkok, Thailand). Dextrans with different molecular mass, monosaccharide standards, Glc, Gal A, PMP, and sodium chloride were bought from Sigma (St. Louis, MO, United States).

Mitochondrial viability stain assay reagent was purchased from Abcam (Cambridge, England, Cat. ab129732). DZ was obtained from Sigma (St. Louis, MO, United States). FBS, trypsin, DMEM, and glucose-free DMEM were bought from Gibco (Carlsbad, CA United States). Griess reagent, inhibitors of phosphoinositide 3-kinase (PI3K, LY29002), ER (tamoxifen), endothelial nitric oxide (eNOS, L-NAME), p-ERK1/2 (U0126) and GR (mifepristone), and the Akt inhibitor IV were purchased from Sigma–Aldrich (St. Louis, MO, United States). All of other reagents were of analysis grade and purchased from the market.

H9c2 cell line derived from rat ventricular muscles was purchased from ATCC (Rockville, MD, United States). The cells were cultured in DMEM filled with 100 μg/mL of streptomycin, 100 U/mL of penicillin and 10% FBS in the normoxic condition (95% air/5% CO_2_) at 37°C. The medium was changed every 2 days, and confluent cells were passaged in a trypsinized way every week. The cells with three to four times of passaging were used in this research.

### High-Speed Counter-Current Chromatography Separation

The ginseng extract was fractionated by using high-speed counter-current chromatography (HSCCC) first. A TBE-1000A HSCCC system produced by Tauto Bio-technique Company (Shanghai, China) with a three-polyterafluoroethylene coil with 1000 mL total capacity was adopted. The β value of the column from internal layer to external layer varied from 0.59 to 0.75. A Waters HPLC instrument equipped with 2425 pump, 2489 UV detector, Flex Inject injector and Fraction Collector III (Waters Corp., Milford, MA, United States) was connected to the HSCCC instrument. The preparation of the diphase solvent system included adding solvents into a separating funnel by the volume ratio and then shaking the solvents in a repeated way at room temperature to fully equilibrate the solvents. After being separated and degassed by sonication, the upper and lower phases stood still overnight prior to usage. After being dissolved in 60 mL of two-phase solvents, the ginseng extract (4.12 g) was centrifuged with a Centrifuge 5810 (Eppendorf, Hamburg, Germany) at 4000 rpm for 15 min, so that supernatant solution can be prepared. Then, the stationary phase, i.e., the upper phase of butanol-water (1:1) was filled in the HSCCC column which was then rotated in the clockwise direction at 800 rpm and filled with the sample solution via an injection loop. Next, the lower layer of butanol-water at a ratio of 1:1 was injected into the HSCCC column thoroughly at 8 mL/min. When the separation time ran to 100 min, the mobile phase was changed to lower phase of ethyl acetate-water (1:1). The effluent in each 5 min was collected as a fraction. Based on the silica gel TLC examination results, the fractions were combined and then freeze dried. The mobile phase of TLC composed of water, methanol and chloroform by ratio of 2:7:13, an UV-254 nm was used to detect TLC. The bioactivity of different HSCCC fractions were examined in H9c2 cardiomyocytes exposed to H/R according to the method described in section “Hypoxia/Reoxygenation and Drug Treatment.”

### Stepwise Ethanol Precipitation

The HSCCC fractions with bioactivity were dissolved in water and further fractionated using stepwise precipitation in 90, 80, and 70% of ethanol solutions successively. Supernatants and precipitates at each precipitation were separated and collected via centrifugation and lyophilization and then examined for their bioactivity by using the method described in section “Hypoxia/Reoxygenation and Drug Treatment.”

### Ion-Exchange Chromatography

The final precipitation in section “Stepwise Ethanol Precipitation” was separated by anion-exchange chromatography on Toyopearl DEAE 650 M (Sigma–Aldrich, St. Louis, MO, United States) which was step wisely eluted with H_2_O, 0.5 M of NaCl, 1.0 M of NaCl, 2.0 M of NaCl and 0.2 M of NaOH to give five fractions, namely, neutral polysaccharides (NP), acidic polysaccharides (AP) 1, AP2, AP3, and AP4. These five fractions were separately dialyzed against 1000 Da sieve (Wako, Osaka, Japan) in water for 24 h, then centrifuged and lyophilized. The bioactivity of the five fractions was examined by using the method described in section “Hypoxia/Reoxygenation and Drug Treatment.”

### Characterization of Polysaccharides

The apparent molecular weight of the active polysaccharides fractions was determined by using HPGPC. The monosaccharide composition was analyzed by HPLC using the reported methods (Xu et al., 2014). The total sugar contents were determined by phenol-H_2_SO_4_ colorimetric method (Dubois et al., 1956). The content of uronic acid was determined by using *m*-hydroxydiphenyl method (Blumenkrantz and Asboe-Hansen, 1973). Protein was determined by Bradford assay.

### Cell Viability

The cell viability was examined with MVS assay. In brief, when cell culture was finished, each well of H9c2 cells with 100 μL of medium was overlaid with 2× mitochondrial viability stain (100 μL), and then the cells were put in the incubator with the normoxic environment (95% air/5% CO_2_) at 37°C for 4 h in the dark. The fluorescent intensity was measured by a Tecan Infinite M200 microplate reader (Tecan, Durham, NC, United States) at excitation 550 nm and emission 590 nm. The cell viability was calculated as the percentage OD value of tested cells over controlled cells.

### Hypoxia/Reoxygenation and Drug Treatment

Hypoxia/reoxygenation treatment was achieved by using a hypoxic chamber (Stem Cell Technologies, United States) to create a challenge of *in vitro* hypoxia environment as described by us previously (Dong et al., 2016). In brief, the cells were firstly cultured in DMEM under normoxic environment (95% air/5% CO_2_) at 37°C overnight, then washed twice with KRB buffer (composition in mM: NaCl 115, KCl 4.7, CaCl_2_ 2.5, KH_2_PO_4_ 1.2, MgSO_4_ 1.2, NaHCO_3_ 24, HEPES 10; pH 7.4) prebalanced with N_2_ at 4°C overnight. Next, 100 μL of KRB and 0.01% (w/v) BSA were added in each well. Then, the cells were placed in the chamber and flushed with pure N_2_ for 5 min at a flow rate of 20 mL/min, all connectors of the chamber were then closed and sealed. The cells with the chamber was then moved into an incubator for a hypoxic culture at 37°C for 3 h. After the hypoxia incubation, KRB in each well was replaced with fresh DMEM, and the cells were incubated for another 4 h in the normoxic condition (95% air/5% CO_2_) at 37°C without chamber.

#### Drug Treatment

The bioactivity of different ginseng fractions was examined with H9c2 cells in a density of 8 × 10^3^/100 μL/well seeded in a 96-well plate or 2 × 10^4^/2 mL μl/well seeded in a 6-well plate. The tested samples were added to the cells 1 h before hypoxia treatment and remained in the whole H/R process. The normal control cells (Normal) were cultured in DMEM in normoxic environment (95% air/5% CO_2_) at 37°C for 8 h in parallel with the manipulation of H/R treatment. To explore the roles of different kinases in the effect of AP1, the cells were pretreated with 40 μM of U0126 (Erk1/2 inhibitor), 10 μM of Akt inhibitor IV (Akt inhibitor), 40 μM of LY294002 (PI3K inhibitor), 10 μmol/L of L-NAME (eNOS inhibitor), 100 μM of tamoxifen (ER inhibitor) and 100 μM of mifepristone (GR inhibitor) (Turner et al., 2000; Khan et al., 2002; Park et al., 2003; Dunn et al., 2009; Buontempo et al., 2011; Todd et al., 2014) for 1 h prior to AP1 (200 μg/mL) treatment.

### Measurement of Lactate Dehydrogenase and Creatine Kinase Activities

After H/R injury, the cell culture medium was collected for the measurement of LDH and CK activities by using commercial assay kits (Roche, Switzerland or Biovision, United States) according the methods recommended by the manufacturer using a spectrophotometer detected at 450 and 660 nm, respectively.

### Measurement of Intracellular Malondialdehyde Level and Superoxide Dismutase Activity

After H/R injury, the cellular protein was extracted and quantified using the bicinchoninic acid (BCA) method. Then, the intracellular protein was used to measure MDA level at 532 nm and superoxide dismutase activity (SOD) activity at 450 nm by using commercial assay kits (Beyotime Institute of Biotechnology, Beijing, China) according the methods recommended by the manufacturer.

### Measurement of Intracellular ROS and Mitochondrial ROS

The intracellular and mitochondrial ROS level was measured with DCFH-DA probe or mitoSOX as previously reported method with modification (Zhang et al., 2015). In brief, after H/R injury, the cell pellets were collected and cleansed with serum-free media. Then, the cells were mixed with serum-free media containing 10 μM DCFH-DA probe (Molecular Probes, Eugene, OR, United States) or 5 μM mitoSOX (Thermo Fisher Scientific, Waltham, MA, United States) and stayed at 37°C in darkness for 30 min with slight agitation every 5 min. Then, the cell pellets were collected and cleansed in PBS for three times, and suspended in 500 μL PBS for flow cytometry analysis on BD FACSAria III (BD Biosciences, Franklin Lakes, NJ, United States). The induced wavelength of the green fluorescence sent from 10,000 cells was documented at 488 or 510 nm. The Flow J software was used to analyze the average intensity of fluorescence.

### Western Blot Analysis

After H/R injury, the cell pellets were collected and cleansed with cold PBS for two times. To determine the release of cytochrome C from mitochondria to cytosol, the extraction of cell cytosolic and mitochondrial fractions were performed using the Cell Mitochondria Isolation Kit (Thermo Fisher Scientific, Waltham, MA, United States). Total cell proteins were extracted by RIPA lysing buffer (1% Nonidet P-40, 1% sodium deoxycholate, 0.5% SDS, 150 mM NaCl, pH 7.5) for 20 min, then centrifuged at 14,000 *g* and 4°C for another 20 min, the supernatants were collected and stored at -80°C. The protein concentration was measured by a Bradford Protein Assay Kit (Bio-Rad, Hercules, CA, United States). Same quantities (40 μg) of protein were loaded and separated on sodium dodecyl sulfate polyacrylamide gel electrophoresis (SDS-PAGE) and then conveyed to a nitrocellulose membrane. The membranes in Tris-glycine buffer were plugged with 5% of skim milk powder in saline [with 0.1% (v/v) Tween-20] buffered with Tris-glycine at room temperature for 1 h, incubated with the primary antibodies of p-JNK, JNK, p-p38, p38, p-ERK, ERK, GR, p-PI3K, PI3K, p-Akt, Akt, inducible NOS (iNOS), cytochrome c, Bcl-2, Bax (Cell Signaling Technology, Boston, MA, United States), ER, p-eNOS, eNOS, β-actin (Santa Cruz Biotechnology, Santa Cruz, CA, United States) and COX IV (Abcam, Cambridge, MA, United States) at 4°C overnight, and then the secondary antibodies conjugated with IRDye (LI-COR Biosciences, Belfast, ME, United States) for 1 h at room temperature. The Odyssey CLx Imaging System (Li-COR Biosciences, Belfast, ME, United States) were used to obtain the band of the antigen-antibody complex and the Odyssey v3.0 software was employed for the densitometric analysis to obtain band intensity.

### Measurement of Nitric Oxide

After H/R injury, the cell culture medium was collected for measuring the level of nitric oxide (NO) by using the Griess reaction according to the reported method (Chen et al., 2012) using a commercial kit of Promega (Madison, WI, United States). Briefly, the cell culture medium (40 μL) was mixed with NADPH (1 mM, 10 μL) and a basic solution (composed of 1.25 mM of glucose-6-phosphate, 0.03 M of PBS, 400 U/L of glucose-6-phosphate dehydrogenase and 200 U/L of nitrate) and stayed at room temperature for 45 min. Then, the Griess reagent (50 μL) added to the mixture and stayed at room temperature in darkness for 20 min. Finally, the absorbance of the mixture was measured at 540 nm. The level of NO was calculated according to the instruction of the kit.

### Apoptosis Analysis With Flow Cytometer

A FITC Annexin V Apoptosis Detection Kit (BD, Franklin Lakes, NJ, United States) was employed to measure the percentage of apoptotic cells according to the manufacturer’s instructions. In brief, the cells exposed to H/R injury were washed with PBS and suspended in 1 × binding buffer containing FITC-labeled Annexin V and propidium iodide. After standing in darkness at room temperature for 15 min, the cell suspension was analyzed on BD FACSAria III (BD Biosciences, Franklin Lakes, NJ, United States). The induced wavelength of the green fluorescence sent from 10,000 cells was documented at 488 nm. The Flow J software was used to analyze the percentage of apoptotic cells.

### Determination of Caspase Activity

The activity of Caspase -3/7 and -9 were measured as described previously (Lu et al., 2010) using a Caspase-Glo assay kit (Promega, Madison, WI, United States). In brief, caspase-3/7 reagent (100 μL/well) or caspase-9 reagent (100 μL/well) was added to the cells after H/R injury and incubated in darkness for 1 h on a shaker at room temperature. Then, the luminescence was determined with a Tecan Infinite M200 microplate reader (Tecan, Durham, NJ, United States).

### Measurement of Mitochondrial Membrane Potential

The MMP was measured using rhodamine 123 (Molecular Probes, Eugene, OR, United States) according to the reported method (Joshi and Bakowska, 2011). In brief, the cells exposed to H/R injury were washed with PBS, added with rhodamine 123 (final concentration 10 μM), and kept at 37°C for 30 min in darkness. The fluorescence of cells cultured in 6-well plate was observed with a DeltaVision Elite Cell Imaging System (Applied Precision Inc., GE Healthcare Company, United States), the luminescence of cells cultured in 96-well plate was determined with a Tecan Infinite M200 microplate reader (Tecan, Durham, NC, United States).

### Determination of ATP Level

The ATP level was determined with an ATP measurement detection assay kit (Abcam, United States) according to the manufacturer’s instructions. Briefly, the cells exposed to H/R injury were incubated with ATP substrate solution (100 μL/well) in darkness for 5 min on a shaker at room temperature. The luminescence of each well was determined with a Tecan Infinite M200 microplate reader (Tecan, Durham, NC, United States).

### Evaluation of Oxygen Consumption Rate

Mitochondrial respiration is a strong indicator of the functional bioenergetics capacity of mitochondria and overall cellular health. OCR is an index of cellular oxygen consumption and can used to reflect the mitochondrial respiration. In this research, OCR was evaluated by XFp Extracellular Flux Analyzer (Seahorse Biosciences, North Billerica, MA, United States) in accordance with a modified protocol as previously described (Duicu et al., 2015). In brief, the cells were seeded in Seahorse XFp cell cultured miniplates in 5000 cells/well and subject to H/R treatment. One day before the experiment, the sensor cartridge of an XFp analyzer was hydrated in the non-CO_2_ incubator at 37°C. The XF assay medium 2.5 M glucose, not buffered and supplied by Seahorse Biosciences was used to replace the incubation medium. To form the baseline rate, the OCR rate was measured at all time and then the metabolism of cells was put in a challenge (for the purpose of changing their bioenergetics profiles) incurred by adding three solutions: 10 μM oligomycin (complex V inhibitor), 2 μM FCCP, and 0.5 μM antimycin A/Rotenone (inhibitors of complex I and III). The wells without cell seeding was used to correct background and normalize the reading of each well to the background plate noise. OCR was normalized for total protein per well and expressed as pmol/min/μg protein.

### Statistic Analysis

One-way ANOVA was used to analyze the statistical significance among different groups with the Graph Pad Prism 6.0 software (Graph Pad Software Inc., La Jolla, CA, United States). *P* < 0.05 was regarded as statistically significant. All measurements were repeated in 3∼6 wells in each experiment, the results were obtained from 3 independent experiments.

## Results

### Bioassay-Guided Separation of Ginseng Extract

**FIGURE 1:**
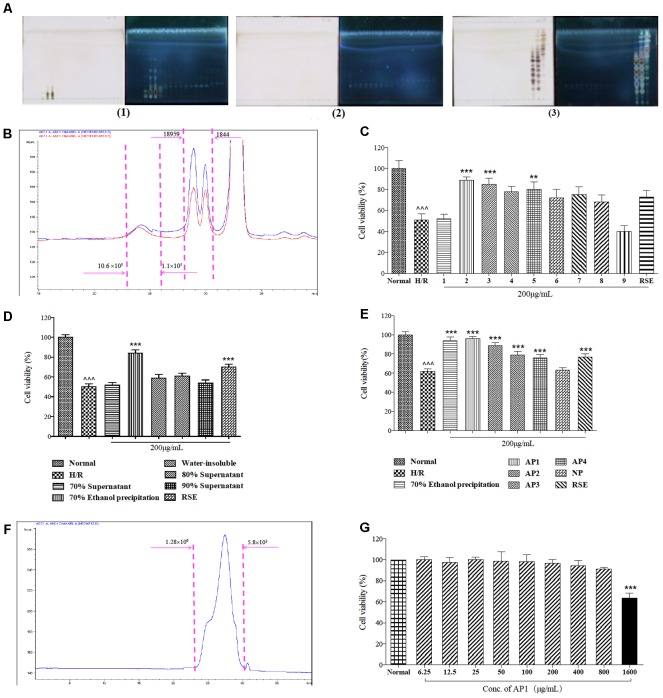
Bioassay guided fractionation of cardioprotective components from ginseng extract. **(A)** The TLC spectrum of the 50 fractions of ginseng extract in HSCCC separation. The TLC spectrum of fraction 1–17 of ginseng extract (1); The TLC spectrum of fraction 18–34 of ginseng extract (2); The TLC spectrum of fraction 35–50 of ginseng extract (3). **(B)** Molecular weight distribution of ginseng extracts 2 and 3. **(C)** Protective effects of ginseng extracts in H9c2 cells exposed to H/R injury. **(D)** Protective effects of fractions of ginseng extract 2/3 in H9c2 cells exposed to H/R injury. **(E)** Protective effects of five fractions of total 70% ethanol precipitates fraction in H9c2 cells exposed to H/R injury. **(F)** Molecular weight distribution of AP1. **(G)** Cytotoxicity of AP1 in H9c2 cells under normoxic conditions. MVS assay was employed to measure cell viability. The results are presented as a percentage of normal group. Data are presented in mean ± SD of three independent experiments. *N* = 3. **(C–E)** ˆ ˆ ˆ *P* < 0.001 vs. Normal, ^∗∗^*P* < 0.01 vs. H/R, ^∗∗∗^*P* < 0.001 vs. H/R, **(G)** ^∗∗∗^*P* < 0.001 vs. Normal.

After HSCCC separation of 4.12 g of ginseng extract, the elution was collected in 48 tubes. After separation, the HSCCC column still had 1000 mL solution, including the lower phase and the upper phase, and such solution was squeezed out due to the high-pressure gasses, so that the last two fractions were obtained. These fractions were combined to nine fractions according to the similarity in their TLC pattern (**Figure 1A**). The total recovery was 94.36%. Besides small polysaccharides (MW < 1800 Da), high polymeric polysaccharides whose molecular weights varying from 1800 Da to 10.6 × 10^5^ Da were also found as the major components (**Figure 1B**).

Next, the cardioprotective effects of these nine fractions were examined. Among them, fractions 2 and 3 were demonstrated to be the most effective fractions (**Figure 1C**) that exhibited identical molecular distribution pattern as revealed by HPGPC. Further stepwise ethanol precipitation of fractions 2/3 mixtures (1:1, 0.74 g) was performed, this offered 4 smaller fractions. Among these four fractions, the precipitate generated from precipitation in 70% ethanol exhibited the strongest activity (**Figure 1D**) and was further subjected onto ion-exchange column chromatography on Toyopearl DEAE 650 M. After isolation, five fractions were collected, and named NP, AP1, AP2, AP3, and AP4. After dialysis and lyophilization, they accounted for 28.96, 39.39, 5.24, 1.37, 1.12% of total 70% ethanol precipitates fraction, respectively. The total recovery was 76.15%. Among them, AP1 was testified as the major fraction, presenting the highest efficacy in preventing cardiomyocytes from death (**Figure 1E**). Thus, AP1 fraction was selected for further study as the effective constituent of ginseng for cardioprotection.

### Chemical Characterization and Cytotoxicity of AP1

Principal components analysis identified that 90.28% of AP1 was carbohydrate composition. Its uronic acid content was 17.93% (w/w), while the protein content was 9.85%. The apparent molecular weight of AP1 was estimated to be 3.8 × 10^4^ (**Figure 1F**). Carbohydrate composition analysis revealed that AP1 was mainly composed of Glc (76.31%), Gal (12.70%) with small amount of Gal A (5.64%), Ara (3.72%), Glc A (1.64%). Based on these results, AP1 was characterized as acidic heteroglycan. Furthermore, the cytotoxicity of AP1 to normal H9c2 cells was tested under normoxic environment. Pretreatment of AP1 at concentrations of 6.25–800 μg/mL for 24 h didn’t cause cytotoxicity to H9c2 cardiomyocytes (**Figure 1G**).

### Cardioprotective Effect of AP1 in H9c2 Cardiomyocytes

**FIGURE 2:**
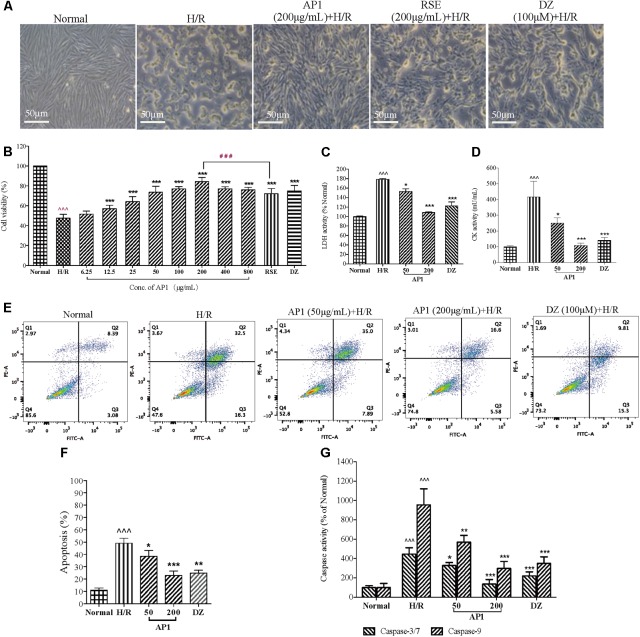
Effect of AP1 on cell viability and apoptosis induced by H/R injury in H9c2 cardiomyocytes. **(A)** Effect of AP1 on cell morphology of H9c2 cells exposed to H/R. 100 μM of DZ was adopted as positive control. The normal control cells were always cultured in DMEM in normoxic environment in parallel with the manipulation of H/R treatment. **(B)** Dose-response relationship of AP1 on cell viability measured by MVS assay. **(C,D)** Effect of AP1 on LDH and CK activities in H9c2 cells exposed to H/R. **(E,F)** Effect of AP1 on the apoptosis of H9c2 cells exposed to H/R. Flow cytometry was used to detect inhibition of H/R-induced cell apoptosis by AP1 under assistance of PI and Annexin V-FITC. Cells in Q4 were living cells, in Q2 were late apoptotic cells, and in Q3 were early apoptotic cells. **(G)** Effect of AP1 on the activity of caspase -3/7 and -9 in H9c2 cells exposed to H/R. The results are presented as a percentage compared to the normal group. Data are presented in mean ± SD of three independent experiments. *N* = 3. ˆˆˆ*P* < 0.001 vs. Normal, ^∗^*P* < 0.05 vs. H/R, ^∗∗^*P* < 0.01 vs. H/R, ^∗∗∗^*P* < 0.001 vs. H/R, ^###^*P* < 0.001 vs. RSE.

The cardioprotective effect of AP1 against H/R injury was investigated in H9c2 cells by morphological observation and MVS assay at first. Normally, H9c2 cardiomyocytes adhered to the plate uniformly in a filamentous shape as reported (Sardao et al., 2009). When exposed to hypoxia for 3 h and followed by reoxygenation for 4 h, the cells became round or irregular shapes, indicating the cytotoxic effect of H/R injury (**Figure 2A**). However, AP1 at 200 μg/mL largely retained the normal shape of cardiomyocytes. The cell viability of H/R group significantly decreased (**Figure 2B**, *P* < 0.001, vs. Normal control). DZ, a mitochondria potassium channel opener (Janjua et al., 2014) that was potent in protecting hearts and cardiomyocytes from I/R injury (Henn et al., 2015), was used as positive control and showed significant protection to the cell at 100 μM (**Figure 2B**, *P* < 0.001, vs. H/R). AP1 pretreatment dose dependently increased the viability of H9c2 cell exposed to 3 h hypoxia and 4 h reoxygenation (**Figure 2B**, *P* < 0.001, vs. H/R). AP1 at even a very low dose (12.5 μg/mL) still produced protective effect. AP1 at 200 μg/mL produced the best protective effect among the dosages examined and was better than the general extract of ginseng RSE (200 μg/mL), showing AP1 is one of the major active constituent of RSE and ginseng. If the dosage further increased to 400 or 800 μg/mL, AP1 although still had protective effect, the potency decreased a little compared with 200 μg/mL, implying the appearance of potential cytotoxicity. Therefore, the highest dosage of AP1 used in other experiment was 200 μg/mL.

The loss of cell membrane integrity caused by H/R injury results in the release of LDH and CK, indicators of myocardium injury and terminal apoptotic cells. AP1 pretreatment dose dependently and remarkably decreased the elevated activities of LDH and CK induced by H/R (**Figures 2C,D**, *P* < 0.05∼0.001). Considering the importance of apoptosis in determining cell death after ischemia/reperfusion, anti-apoptotic effect of AP1 was assessed. Annexin V/PI double staining showed that the apoptosis of H/R group increased sharply (**Figure 2E**, *P* < 0.001 vs. Normal). However, AP1 and DZ remarkably reversed this, showing by significant attenuation of H/R induced apoptosis (**Figure 2F**, *P* < 0.05∼0.001 vs. H/R). Caspase-3/7 and caspase-9 are important apoptotic indicators in the apoptotic pathway relied on mitochondria (Li et al., 1997; Porter and Janicke, 1999). Thus, the effect of AP1 on the activities of caspase-3/7 and caspase-9 in H9c2 cells with H/R injury was examined. H/R induced significant increase in the activities of caspase-3/7 and caspase-9 in H9c2 cells exposed to H/R (**Figure 2G**, *P* < 0.001, vs. Normal). However, AP1 at 50 and 200 μg/mL and DZ at 100 μM reduced the activities of caspase-3/7 and caspase-9 (**Figure 2G**, *P* < 0.05∼0.001, vs. H/R), showing that the caspase-dependent apoptotic pathway plays a role in the cardioprotective effect of AP1.

These results suggested that AP1 had obvious cardioprotective effect to H9c2 cell against H/R induced injury through the reduction of apoptosis.

### AP1 Protected H9c2 Cells From H/R Injury by Reduction of Oxidation and Reactive Oxygen Species Mediated JNK/p38-MAPK Activation

**FIGURE 3:**
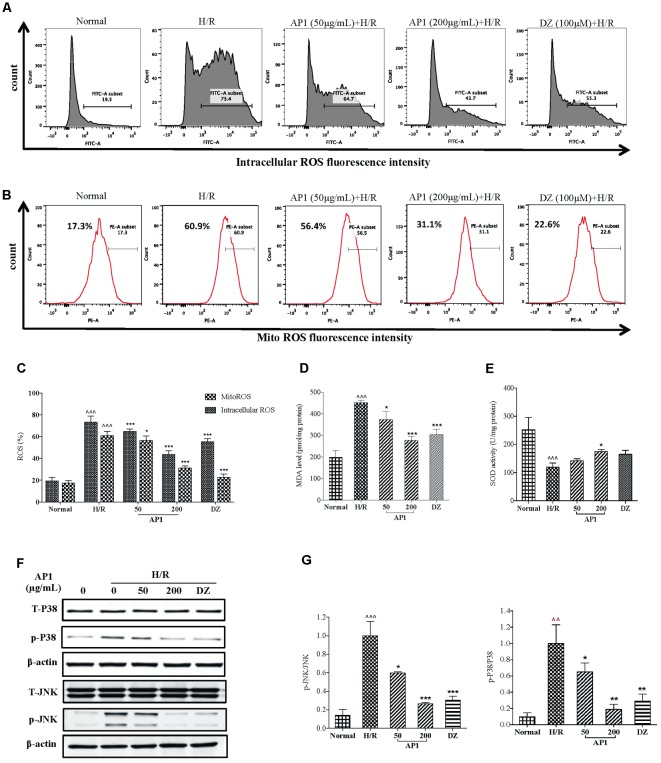
Effect of AP1 on oxidation and ROS production in H9c2 cells exposed to H/R. **(A)** Effect of AP1 on intercellular ROS formation. **(B)** Effect of AP1 on mitochondrial ROS formation. **(C)** Quantification of intracellular and mitochondrial ROS. **(D)** Effect of AP1 on MDA contents. **(E)** Effect of AP1 on SOD activity. **(F,G)** Effect of AP1 on phosphorylation of p38 and JNK. Data are presented in mean ± SD of three independent experiments. ˆ ˆ *P* < 0.01 vs. Normal, ˆˆˆ*P* < 0.001 vs. Normal, ^∗^*P* < 0.05 vs. H/R, ^∗∗^*P* < 0.01 vs. H/R, ^∗∗∗^*P* < 0.001 vs. H/R.

Evidences suggest that overproduction of ROS impairs mitochondrial function, which leads to further reperfusion toxicity (Takano et al., 2003). Considering cellular ROS release plays an important role in the activation of the preconditioning protection in the cardiovascular system (Becker, 2004), the effect of AP1 on the intracellular level of ROS was examined so as to inspect the association between the release of intracellular ROS and cardioprotection of AP1. As shown in **Figures 3A,C**, H/R induced significant release of intracellular ROS, showing by the increased fluorescence intensity (*P* < 0.001 vs. Normal). However, the intracellular ROS levels dramatically dropped down by the pretreatment of AP1 in a dose dependent manner (*P* < 0.05∼0.001 vs. H/R). In addition, since mitochondrial ROS (mitoROS) is the most important source of the intracellular ROS (Kalogeris et al., 2014), to understand the effect of AP1 on the mitoROS production, we compared the mitoROS production of H9c2 cell underwent H/R with or without AP1 treatment. The results showed that H/R caused the elevation of mitoROS production, while AP1 pretreatment suppressed this elevation (*P* < 0.05∼0.001 vs. H/R) (**Figures 3B,C**).

In addition, the concentration of myocardial MDA, a byproduct of ROS reflecting myocardial oxidative damage (Gago-Dominguez et al., 2002), and the activity of SOD that is a main source of ROS scavenger (Nishikawa et al., 2000) were also evaluated. As shown in **Figures 3D,E**, H/R significantly induced increase of MDA level and decrease of SOD activity. Nevertheless, AP1 treatment dose dependently recovered the change of MDA level and SOD activity caused by H/R (*P* < 0.05∼0.001, vs. H/R).

As an early signal of apoptosis, ROS also directly involves in the activation of JNK and p38 signaling (Benhar et al., 2001; Shen and Liu, 2006), which play an indispensable role in oxidative stress induced apoptosis (Xia et al., 1995). Therefore, the change of these signaling proteins in H/R injury was investigated. As shown in **Figures 3F,G**, H/R induced phosphorylation of p38 and JNK without influencing the total p38 and JNK; while AP1 pretreatment significantly inhibited the phosphorylation of JNK and p38 induced by H/R in a dose-dependent manner (*P* < 0.05∼0.001, vs. H/R).

These results suggested that AP1 protected H9c2 cells from H/R injury by reduction of oxidation and ROS mediated JNK/p38-MAPK activation.

### AP1 Effectively Alleviated Mitochondrial Dysfunction Caused by H/R Injury

**FIGURE 4:**
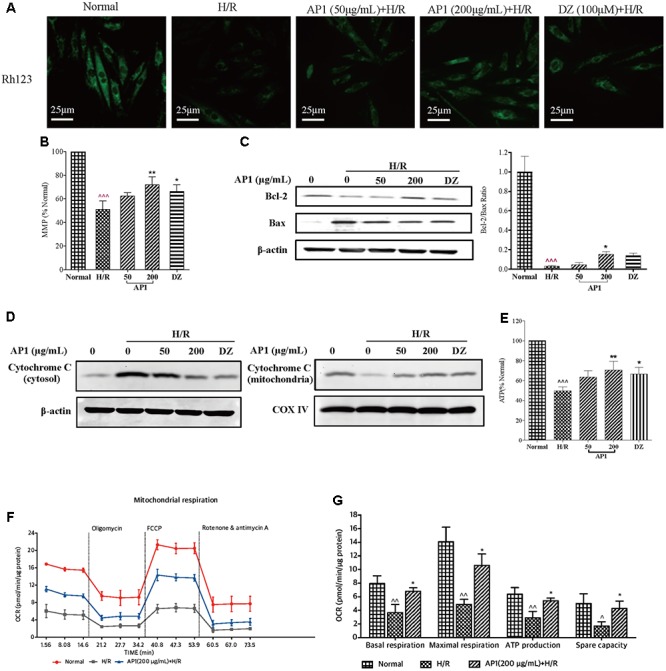
Effect of AP1 on the mitochondrial functions of H9c2 cell exposed to H/R. **(A,B)** Effect of AP1 on MMP (mitochondrial membrane potential). **(C)** Effect of AP1 on Bcl-2/Bax ratio. **(D)** Effect of AP1 on cytochrome c in mitochondria and the cytoplasm. COX IV was used as an internal loading control for mitochondrial proteins. **(E)** Effect of AP1 on ATP generation. **(F,G)** Effect of AP1 on OCR. The results are presented as a percentage compared to the normal group. Data are presented in mean ± SD of three independent experiments. ˆ *P* < 0.05 vs. Normal, ˆ ˆ *P* < 0.01 vs. Normal, ˆ ˆ ˆ *P* < 0.001 vs. Normal, ^∗^*P* < 0.05 vs. H/R, ^∗∗^*P* < 0.01 vs. H/R, ^∗∗∗^*P* < 0.001 vs. H/R.

Mitochondrial dysfunction is a major consequence of H/R injury and MMP is a direct indicator of mitochondrial respiration efficiency (Petrosillo et al., 2009). Therefore, the MMP of H9c2 cell was measured. As shown in **Figure 4A**, H/R injury caused a significantly decreased uptake of Rh123, showing the dissipation of MMP (**Figure 4B**, *P* < 0.001 vs. Normal). Interestingly, the loss of MMP was ameliorated by pretreatment of AP1 (**Figures 4A,B**, *P* < 0.01 vs. H/R), implying that AP1 preserved the mitochondrial function of cardiomyocytes exposed to H/R.

Furthermore, according to earlier researches, the potential of cellular apoptosis is decided by expression of Bcl-2 and Bax (Yang et al., 1997). Besides, the Bcl-2 to Bax ratio is important for maintaining the integrity of mitochondrial membrane. Therefore, the expression of Bcl-2 and Bax in H9c2 cells exposed to H/R treatment was examined. The results showed (**Figure 4C**) that H/R treatment remarkably reduced the expression of Bcl-2 but increased the expression of Bax, causing a significant decrease of Bcl-2 to Bax ratio. However, AP1 at 200 μg/mL slightly reversed the decease of Bcl-2 and increase of Bax, causing an increase of Bcl-2 to Bax ratio in H9c2 cells exposed to H/R treatment (*P* < 0.05 vs. H/R).

The activation of intrinsic apoptosis is imitated by the release of cytochrome C from mitochondria to cytosol, therefore the level of cytochrome C in cytoplasm was also determined. The results (**Figure 4D**) showed that H/R treatment caused a remarkable release of cytochrome C to cytoplasm in H9c2 cells but decreased its level in mitochondria. However, pretreatment with AP1 dose dependently and significantly reduced the release of cytochrome C. The positive control drug DZ at 100 μM also showed the same effect. In addition, the generation of intracellular ATP levels was evaluated. The exposure of H9c2 cells to H/R significantly inhibited ATP generation (**Figure 4E**, *P* < 0.001 vs. Normal). This inhibition was reversed by AP1 and DZ (*P* < 0.05∼0.01 vs. Normal).

To further interrogate the mitochondrial metabolic state of H/R injured H9c2 cells, the mitochondrial respiration was evaluated by measuring OCR in Seahorse XFp Extracellular Flux Analyzer. As shown in **Figures 4F,G**, the basal respiration (OCR before addition of oligomycin), ATP production (the decrease in OCR following addition of oligomycin), maximal respiration (OCR after addition of FCCP), spare respiratory capacity, were significantly lower in the H/R injured cells than in the normal group (*P* < 0.01), indicating that H/R decreased the energetic demand under baseline conditions, ATP production, maximal capacity of respiratory oxidation, and cell flexibility of H9c2 due to damage of mitochondria. However, AP1 reversed these changes caused by H/R (*P* < 0.05), indicating that AP1 reserved the mitochondrial respiration in H9c2 cardiomyocytes exposed to H/R probably by protecting mitochondria from damage or dysfunction due to H/R.

### AP1 Offered Cardioprotection Against Cell Death via RISK Signaling Pathway

**FIGURE 5:**
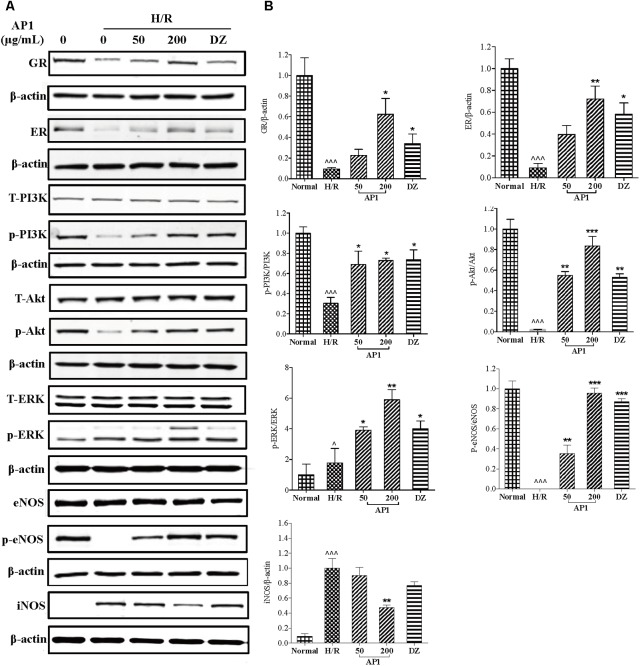
Effect of AP1 on GR, ER/RISK/eNOS pathway of H9c2 cell exposed to H/R. **(A)** Western-blot analysis was performed by the antibody specific to GR, ER, ERK1/2 or phosphorylated ERK1/2, PI3K or phosphorylated PI3K, Akt or phosphorylated Akt, eNOS or phosphorylated eNOS and iNOS. **(B)** Quantitative analyses for protein expression. Data are presented in mean ± SD of three independent experiments. ˆ *P* < 0.05 vs. Normal, ˆˆˆ*P* < 0.001 vs. Normal, ^∗^*P* < 0.05 vs. H/R, ^∗∗^*P* < 0.01 vs. H/R, ^∗∗∗^*P* < 0.001 vs. H/R.

Modulation of RISK pathway (consisting of PI3K/Akt and ERK1/2) by both ischemic preconditioning and postconditioning confers powerful protection to heart exposed to ischemia/reperfusion insult (Davidson et al., 2006; Hausenloy and Yellon, 2007; Kalakech et al., 2014). Our previous research had showed that stimulation of RISK pathway by ginseng extract through GR and ER protected heart from I/R injury in an eNOS dependent manner (Zhou et al., 2011). In this research, the role of GR, ER, RISK pathway, NOS, and NO in the effect of AP1 was also examined. As shown in **Figure 5**, H/R significantly suppressed the expressions of GR and ER (*P* < 0.001 vs. Normal). However, AP1 pretreatment remarkably and partially restore the expressions of GR and ER in H/R injured cells (*P* < 0.05∼0.01 vs. H/R). Similarly, positive control drug DZ also partially reversed the expressions of GR and ER (*P* < 0.05∼0.01 vs. H/R). **Figure 5** shows that H/R didn’t change the expression of total PI3K, Akt, ERK1/2, eNOS, but significantly inhibited the phosphorylation of PI3K, Akt, and eNOS and significantly increased the expression of iNOS and phosphorylation of ERK1/2 (*P* < 0.05∼0.001); while AP1 pretreatment remarkably restored the phosphorylation of PI3K, Akt, and eNOS, further enhanced the phosphorylation of ERK1/2 in H/R injured cells, and suppressed the expression of iNOS (*P* < 0.05∼0.001 vs. H/R). This mechanism of AP1 is similar to that of ginseng extract RSE, implying that AP1 is a major constituent responsible for the cardioprotective effect of ginseng.

### Cardioprotective Effect of AP1 Is Dependent on GR, ER, ERK1/2, PI3K and Akt

**FIGURE 6:**
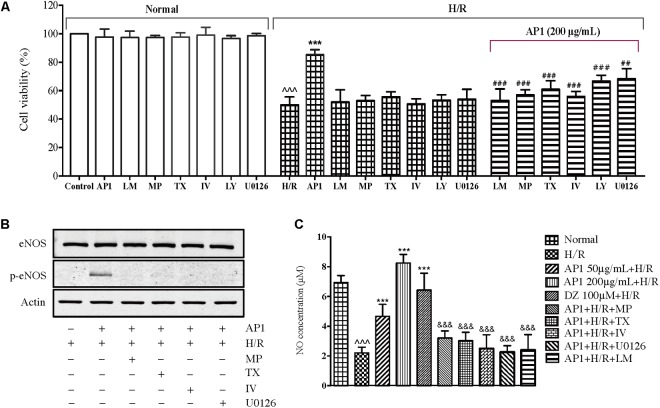
Influence of different pharmacological inhibitors on the effect of AP1 on H9c2 cell exposed to H/R. **(A)** The H9c2 cells were cultured with or without mifepristone (MP, 100 μM), tamoxifen (TX, 100 μM), L-NAME (LM, 10 μmol/L), LY294002 (LY, 40 μM), Akt inhibitor IV (IV, 10 μM), U0126 (40 μM) first, then treated with or without 200 μg/mL AP1, finally exposed to H/R injury. The normal group was always maintained in normoxic condition. Cell death was assessed by MVS assay. **(B,C)** Effects of signaling inhibitors on eNOS protein level and NO production in H/R-stimulated H9c2 cells. Proteins were subjected to immunoblot analysis using antibodies specific to eNOS or p-eNOS **(B)**. After incubation, nitrite in the culture supernatants was determined by a Griess assay **(C)**. The results are presented as a percentage compared to the normal group. Data are presented in mean ± SD of three independent experiments. ˆˆˆ*P* < 0.001 vs. Normal, ^∗∗∗^*P* < 0.001 vs. H/R, ^##^*P* < 0.01 vs. AP1+H/R, ^###^*P* < 0.001 vs. AP1+H/R, ^&&&^*P* < 0.001 vs. AP1 (200 μg/mL + H/R).

The above research suggested that AP1 may act on GR and ER to activate RISK pathway and eNOS and suppress iNOS thus protect cardiomyocytes from H/R injury. To confirm the role of these molecules in the effect of AP1, the cells were pretreated with the inhibitor of NOS (L-NAME), ERK1/2 (U0126), Akt inhibitor IV, PI3K (LY294002), ER (tamoxifen) or GR (mifepristone), and then the effect of AP1 on cell viability was examined. As shown in **Figure 6A**, these inhibitors didn’t change the cell viability of normal H9c2 cells and H/R treated cells, indicating that these inhibitors have no significant influence on the growth and cellular activities of H9c2 cell under normoxic and H/R conditions. However, these inhibitors significantly abolished the protective effect of AP1 on H9c2 cell (*P* < 0.01∼0.001 vs. AP1 alone in H/R insulted cells), implying that the cardioprotective effect AP1 requires the participation of eNOS, ERK1/2, Akt, PI3K, ER and ER.

### Effects of AP1 on NO Production

Nitric oxide is an important factor in affecting the physiology and pathology of the cardiovascular system (Loscalzo and Welch, 1995; Burwell and Brookes, 2008; Shumilova et al., 2014). The above research showed that eNOS is indispensable to the cardioprotective effect of AP1, the NO level is therefore determined as well. As shown in **Figure 6C**, H/R induced significant decrease of NO production in H9c2 cells (*P* < 0.05 vs. Normal), agreeing the decreased phosphorylation of eNOS (**Figure 5**). AP1 and DZ pretreatments recovered the production of NO in H9c2 cells exposed to H/R injury. Interestingly, the change of NO level seems in parallel with the cell viability (**Figure 6C**), i.e., the higher the NO level the larger the cell viability. However, L-NAME (NOS inhibitor) completely suppressed the effect of AP1 on NO production, which is accompanied with the completely loss of protection to H9c2 cells (**Figure 6A**), implying that eNOS dependent NO production is dispensable to the cardioprotective effect of AP1. To further confirm this result, we also investigated whether AP1-induced eNOS activation and NO production were inhibited by pretreatment with GR, ER, PI3K/Akt and ERK inhibitors. The results showed that the pretreatment of these inhibitors for 1 h markedly reduced AP1 induced eNOS phosphorylation and NO production (**Figures 6B,C**), consistent with the effect of NOS inhibitor on NO production. These results support that AP1 induces eNOS activation and NO production via GR and ER dependent RISK signaling pathway in H9c2 cells.

## Discussion

In the present study, we descripted that an acid polysaccharides of ginseng AP1, one of the main constituents presenting in ginseng, maintained the mitochondrial function of cardiomyocytes under H/R and down-regulated the activity of LDH and CK, and thus protected cardiomyocytes from H/R injury. AP1 also inhibited the formation of MDA and ROS during the process of reperfusion injury by effectively increasing the activity of SOD, indicating that ginseng polysaccharides can protect cardiomyocytes from free radical injury. Since mitochondrial apoptosis is a major cause of ischemia-reperfusion damage, we examined the effect of ginseng polysaccharides on mitochondrial-related apoptosis pathways. The results demonstrated that ginseng polysaccharides maintained the MMP, increased ATP generation, blocked the releasing of cytochrome C, further inhibited the cleavage of Caspase-3 and 9, and finally counteracted apoptosis induced by ischemia-reperfusion. Meanwhile, ginseng polysaccharides increased the OCR to maintain the energy conservation. Ginseng polysaccharides also induced the expressions of GR and ER which further activated RISK pathway and acted as the main contributor in regulating the viability of cardiomyocytes suffered reperfusion injury. Finally, ginseng polysaccharides regulated endothelial function by increasing eNOS expression, which also greatly contributed to stabilize myocardium environment, reduced cell death, and protected myocardium from injury.

Traditional Chinese medicine has a history of 1000s of years and made significant contributions to the health and well being of the people. Many of TCM have been studied extensively and identified to be safe and effective in treating IHD (Chan, 1995). Among these, ginseng is the most outstanding one. Emerging studies have demonstrated that whole ginseng extract is capable to alleviate cardiomyocytes injury caused by ischemic reperfusion in *in vivo* and *in vitro* models (Maffei Facino et al., 1999; Wang et al., 2007; Ahn et al., 2011; Zhou et al., 2011). To better delineate the ingredients responsible for these findings, we isolated the active compounds from ginseng extract by bioassay-guided fractionation. Intriguingly, AP1 was characterized as an acid polysaccharides of ginseng extract, which appears to be the most effective component of ginseng responsible for cardioprotection than the whole ginseng extract in treating H/R-induced cell damage in H9c2 cells. To the best of our knowledge, this is the first study to show that an acid ginseng polysaccharides exerts a cardioprotective effect via activation of RISK pathway and subsequent inhibition of mitochondria-dependent apoptotic pathway. Therefore, this study implies that RISK and mitochondrial apoptosis pathways may serve as a pharmacological target of ginseng in protecting heart, and that AP1 might be a hopeful candidate for the prevention of IHD, particularly for myocardial ischemic reperfusion.

Oxidative stress plays a vital role in the pathophysiological mechanism of ischemic reperfusion (Maulik et al., 1998; Ferrari et al., 2004). Ischemic reperfusion impairs the balance of ROS generation and elimination, and results in oxidative stress and reperfusion (Carpi et al., 2009). ROS can induce cardiomyocyte apoptosis through mitochondria-dependent apoptosis pathway (Circu and Aw, 2010). On the one hand, ROS can damage the mitochondria membrane potential and mitochondrial outer membrane integrity, causing release of apoptogentic proteins into the cytoplasm. The release of cytochrome c-mediated apoptotic enzyme cascade, which triggers the execution of apoptosis in mitochondria (Simon et al., 2000). On the other hand, in response to oxidative stress, JNK and P38 are one of the large downstream cascades of ROS signaling that closely related to cell survival, apoptosis and proliferation (Benhar et al., 2001; Huang et al., 2008; Zhang et al., 2011). Activated JNK and P38 can trigger pro-apoptotic Bax expression, while deactivating pro-survival Bcl-2 protein, thereby stimulating mitochondria-dependent apoptosis (Kim et al., 2006). Therefore, targeting ROS accumulation and mitochondrial dysfunction will be promising for prevention of I/R.

In the present study, we demonstrated that the damage caused by I/R injury can be effectively prevented and reversed by medicinal preconditioning with AP1 through activating the RISK pathway, a classic myocardial protective mechanism consisting of PI3K/Akt and Erk1/2 signaling pathways (Davidson et al., 2006; Hausenloy and Yellon, 2007; Kalakech et al., 2014). In RISK pathway after activating by GR, it induces the phosphorylation of eNOS, and subsequently causes the surge of cardioprotective NO production (Zhou et al., 2011). NO is directly implicated in many cardiovascular syndromes, including vasoconstriction, platelet activation, vasodilation, cell apoptosis, and oxidative stress, etc. (Dinerman et al., 1993; Cannon, 1998; Gao et al., 2002; Casas et al., 2004; Schulz et al., 2004). Among those functions, two aspects were proved to be closely associated with cardioprotective ability. Its anti-oxidative potential as free radical scavenger was the most important one, which was supported by an array of experimental facts: NO can effectively protect the myocardium against free-radical injury (Takano et al., 1998; Hung and Kao, 2004). Additionally, the close relationship between NO and mPTP opening was another important aspect (Hausenloy et al., 2005; Davidson et al., 2006). A serial consequence of mPTP opening including mitochondrial swelling, oxidative phosphorylation decoupling and ATP consumption can be inhibited by NO (Hausenloy et al., 2004), resulting in the enhancement of mitochondrial respiration and mitochondrial protection.

Based on our results, AP1 protected cardiomyocytes by up regulating GR or ER and further trigger the cascade of RISK/eNOS/NO pathways to neutralize ROS generation during H/R process, acting as a mimic of ischemic preconditioning. AP1 also stabilized MMP and maintained ATP supply to keep the mitochondrial membrane intact. Subsequently, cytochrome c release was reduced, and caspase cascade was deactivated, which finally resulted in the recovery of mitochondrial respiration and prevention of mitochondria-dependent apoptosis.

Although the current research demonstrated the cardioprotective effect of AP1 and also showed that this effect is related to the regulation of mitochondrial metabolism and activation of RISK pathway in hypoxia/reoxygenized H9c2 cells, there are still some limitations and will be resolved in future research. For example, it is not clear whether this effect of AP1 can be repeated in animal models and if other mechanisms involve in this effect, therefore, mice with left anterior descending coronary artery ligation can be a powerful model to address the *in vivo* effects and therapeutic potential of AP1 treatment. In addition, although the use of pharmacological inhibitors of GR, ER, PI3K/Akt, and ERK supported the involvements of RISK pathway in the effect of AP1, knock down experiments in *in vitro* cultured cells or knock out mice model would definitely further confirm their roles in this regard. It is also interested to know whether repeated dosing could give a better protection to cardiomyocytes and/or animal hearts undertaken ischemia/reperfusion injury insult. Furthermore, the influence of AP1 on other cell types involved in the pathogenesis of IHDs, such as endothelial cells and macrophages, will be investigated in future research to elaborate the action mechanisms of AP1 from a broader view. With the results from these researches, we could have a better and comprehensive understanding to the efficacy of AP1 on IHDs.

## Conclusion

**FIGURE 7:**
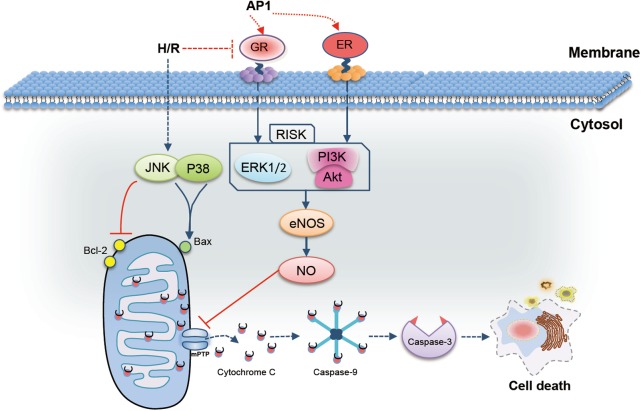
Possible mechanism of the cardioprotective effect of AP1 in H9c2 cells exposed to H/R injury. AP1 induced the expression of GR and ER, which further activated reperfusion injury salvage kinase (RISK, including PI3K/Akt and EEK1/2) pathway. Phosphorylated PI3K/Akt and ERK in turn up-regulated the expression level of eNOS, and further increased NO generation. In addition, AP1 also inhibited the phosphorylation of JNK and p38 and in turn enhanced the ratio of Bcl-2/Bax, which ultimately inhibited the mitochondria-dependent apoptosis pathways to abolish cell death.

An acidic polysaccharides fraction (AP1) derived from ginseng extract with cardioprotective function was discovered for the first time. Mechanistic studies demonstrated that the cardioprotective effect of AP1 involves receptor-mediated activation of RISK/eNOS/NO pathway and modulation of mitochondrial function and metabolism for the first time (**Figure 7**). Although the ginseng polysaccharide is frequently neglected in cardiovascular study, its potential as a novel natural food supplements for the prevention of IHD can be explored further in future.

## Author Contributions

HZ, PL, and LL designed the experiment. Y-HZ, Q-BH, G-TD, R-QY, X-CR, and J-XL performed the experiments. Y-HZ, Q-BH, and HZ analyzed the data. Y-HZ and HZ wrote the manuscript.

## Conflict of Interest Statement

The authors declare that the research was conducted in the absence of any commercial or financial relationships that could be construed as a potential conflict of interest.

## Abbreviations

AP1: acidic polysaccharides fraction of ginseng
Bax: Bcl-2-associated X protein
Bcl-2: B-cell lymphoma 2
CK: creatine kinase
DMEM: dulbecco’s modified Eagle’s medium
DZ: diazoxide
eNOS: endothelial nitric oxide synthase
ER: estrogen receptor
ERK1/2: extracellular signal-regulated kinases
FBS: fetal bovine serum
Gal A: D-galacturonic acid monohydrate
ginseng: Panax ginseng
Glc: D-glucose
GR: glucocorticoid receptor
H/R: hypoxia/reoxygenation
HPGPC: high performance gel filtration chromatography
HPLC: high performance liquid chromatography
HSCCC: high speed counter current chromatography
IHD: ischemic heart diseases
iNOS: inducible nitric oxide synthase
JNK: c-Jun N-terminal kinases
KRB: Krebs-Ringer Bicarbonate
LDH: lactate dehydrogenase
MDA: malondialdehyde
MMP: mitochondrial membrane potential
MVS: mitochondrial Viability Stain
NO: nitric oxide
OCR: oxygen consumption rate
p-eNOS: phosphorylated endothelial nitric oxide synthase
p-ERK1/2: phosphorylated extracellular signal-regulated kinases
p-JNK: phosphorylated c-Jun N-terminal kinases
PMP: 1-pheny-3-methy-5-pyrazolne
RISK: reperfusion injury salvage kinase
ROS: reactive oxygen species
SOD: superoxide dismutase
TCM: traditional Chinese medicine
TFA: trifluoroacetic acid
TLC: thin layer chromatography.
